# Maternal Antenatal Bereavement and Neural Tube Defect in Live-Born Offspring: A Cohort Study

**DOI:** 10.1371/journal.pone.0163355

**Published:** 2016-09-29

**Authors:** Katja Glejsted Ingstrup, Chun Sen Wu, Jørn Olsen, Ellen Aagaard Nohr, Bodil Hammer Bech, Jiong Li

**Affiliations:** 1 Section for Epidemiology, Department of Public Health, Aarhus University, Aarhus C, Denmark; 2 Research Unit for Obstetrics and Gynecology, Institute of Clinical Research, University of Southern Denmark, Odense C, Denmark; 3 Department of Epidemiology, Fielding School of Public Health, University of California, Los Angeles, CA, United States of America; 4 Department of Clinical Epidemiology, Aarhus University Hospital, Aarhus N, Denmark; Pennington Biomedical Research Center, UNITED STATES

## Abstract

**Background:**

Maternal emotional stress during pregnancy has previously been associated with congenital neural malformations, but most studies are based on data collected retrospectively. The objective of our study was to investigate associations between antenatal maternal bereavement due to death of a close relative and neural tube defects (NTDs) in the offspring.

**Methods:**

We performed a register-based cohort study including all live-born children (N = 1,734,190) from 1978–2008. Exposure was bereavement due to loss of a close relative from one year before conception to the end of the first trimester of pregnancy. The outcome was NTDs in the offspring according to the International Classification of Disease. We used multivariate logistic regression to estimate prevalence odds ratios (ORs).

**Results:**

A total of 2% children were born to mothers who lost a close relative prenatally. During 30 years of follow-up, 1,115 children were diagnosed with any NTDs: spina bifida (n = 889), anencephaly (n = 85) and encephalocele (n = 164). And 23 children were diagnosed with two types of NTDs. Overall, when comparing bereaved mothers to non-bereaved mothers, no significant increased prevalence of NTDs in the offspring was seen (OR = 0.84; 95% confidence interval: 0.52–1.33).

**Conclusion:**

Overall maternal bereavement in the antenatal period was not related to NTDs in liveborn offspring.

## Introduction

Malformations in the nervous system are the second most common congenital anomaly in the world with a prevalence of 18.8 cases per 10,000 births in Europe with 7.5 cases being neural tube defects. [1] Children born with a congenital malformation in the nervous system decreased from 4.5% of all congenital malformations in 1994 to 3.1% in 2005 in Denmark [2], probably due to prenatal screenings.

The development of the neural tube takes place in the embryo in week 3–4 after conception, and primary neurulation is responsible for generating the entire neural tube. The neural plate forms 18 days after conception, and the edges of the plate move upward and fold along the midline to form the neural groove on day 19, then a tube, which is open at both ends by day 23. Finally, on day 26 and 28 of gestation, the neural tube closes at the rostral and caudal ends respectively. [3] There are at least two theories of the closing procedures in humans; one is a single site “zipper-like” closure,[4] another a multisite closure [5]. The most common types of neural tube defects are spina bifida (occulta/cystica/aperta), and anencephaly. [6] Spina bifida occurs when a bone defect of the spine let meninges, cerebrospinal fluid and/or nervous tissue form a sack outside the normal curves of the spine. Anencephaly is the absence of the brain and skullcap either partial or total. [6] Previous studies show about 37–43% of the NTDs cases to be strictly isolated,[7] with the most common type of additional malformation being in the muscle and skeletal system (50%) [8].

The etiology of NTDs is probably complex and largely unknown, but causes are believed to be both of genetic and environmental nature. In the early 1990s, randomized controlled trials documented folic acid supplementation to be an important preventable factor for NTDs recurrence risk [9] and later shown also for mothers with no history of NTDs. [10] Defects in the homocysteine metabolism may lead to accumulation of homocysteine, which have teratogenic effects on the developing embryo. [11] [12] Medicines and genes that interfere with the metabolism of folate have been much studied in connection with NTDs, [13–16] emotional stress such as maternal antenatal bereavement is however also of interest as a potential risk factor for neural tube defects. Previous human studies indicate a higher prevalence of NTDs among offspring born to mothers who experienced emotional stress in the prenatal period or up to one year before conception. Maternal emotional stress may lead to a higher concentration of stress-related hormones like glucocorticoids in the mother, which may transfer to the fetus and affect organ development. [17, 18] An elevated level of homocysteine has been measured in women with emotional stress, [19–21] and also elevated homocysteine has been found in plasma [22] and amniotic fluid [23] of women having pregnancies affected with NTDs compared to women with pregnancies unaffected by NTDs. [24] However, other mechanisms are also possible such as a change in lifestyle (increased alcohol [25] intake or smoking [26] or insufficient intake of folic acid due to the stress). Studies from California, Denmark, Italy and China found a 1.5–4 fold increased prevalence of NTDs in the offspring if the mother before or during pregnancy experienced high emotional stress compared to mothers who did not experience emotional stress in that period. [27–33] Most studies focusing on NTDs are case-control studies based on retrospectively collected stress data. [27–29, 31, 32] The aim of the current study was to investigate if exposure to maternal antenatal bereavement due to death of a close relative up to one year before pregnancy and during the first trimester of pregnancy increased the prevalence of NTDs in the live born offspring in a large register-based study with prospective data on exposure.

## Methods

The current population-based cohort study was part of the Nordic Perinatal Bereavement Cohort.[34] Data was retrieved from different Danish National Registers. In Denmark, every child born alive since 1968 and every person living in Denmark have been assigned a unique identification number in the Danish Civil Registration System. [35] We linked the mother to her relatives, the biological father of the child and the child through this identification number (including maternal half siblings). We used information from the Danish Death Registry [36] and the Danish Medical Birth Registry [37] to obtain the timing and cause of death of the maternal relatives according to the conception and pregnancy of the index child.

We included live-born children in Denmark from 1978–2008 with information available in the Danish Medical Birth Registry (N = 2,085,521). [37] We excluded mothers who had an intake of anti-epileptic drugs during pregnancy (n = 10,232), mothers with gestational diabetes or diabetes (n = 17,876) and mothers who previously have had a child with a neural tube defect (n = 269) due to their increased risk of having another child with the same or similar condition. Moreover, we had many missing variables on non-Danish original mothers, and we therefore excluded these mothers (n = 309,798). We restricted our exposure window to one year before conception and during the first trimester and excluded mothers only exposed during the second and third trimester (n = 13,156). Our final study population was 1,734,190 children.

### Exposure

Exposure was defined as exposure to antenatal bereavement due to death of a close relative. We included relatives on the mother’s side including her parents, siblings and children and also the registered (biological) father of the unborn child. Our exposure window was one year before conception to the end of the first trimester. We used three approaches to categorize maternal bereavement: Kind of relative, types of death, and timing of death. We hypothesized that bereavement during the time of organogenesis to have the highest impact on the risk of developing NTDs in the offspring and sudden death in older children and fathers to be the most severe stressors.

### Outcome

Our outcome was NTDs in the offspring. We obtained information about the outcomes from the Danish National Patient Register. [38] All diagnosis was until 1994 defined after the International Classification of Disease (ICD)-8 codes (740.00–743.99) and from 1995 after the ICD-10 codes (Q00–Q07). The codes used for NTDs were as follows; spina bifida ICD-8 (741.00–741.99) and ICD-10 (Q059A), for anencephaly ICD-8 (740.99) and ICD-10 (Q00.0), for encephalocele ICD-8 (743.00, 743.08, and 743.09) and ICD-10 (Q01.0–Q01.9). There were 23 children that had more than one diagnosis of NTD.

### Statistical analysis

We used multiple logistic regression to investigate the association between exposure to antenatal bereavement and NTDs in the offspring. We used all types of NTD diagnoses (spina bifida, anencephaly and encephalocele) as an outcome. Risks of the outcomes were estimated by a prevalence odds ratio (OR) at birth indicating a risk of getting a live-born child with NTDs and not the risk among exposed fetuses, and we use the term risk to facilitate readability. We first compared risk of outcomes in children of mothers who had experienced any kind of bereavement from any type of relative at any time point in the exposure window to children of mothers who had experienced no bereavement. We then stratified according to the type of bereavement (sudden death or other deaths of a relative), into type of relative (death of a child or death of other relatives) and different time windows (during 12–7 month or 6–0 month before conception or during the first trimester) and compared all the different exposure groups to mothers who had experienced no bereavement at all. We had insufficient data to do a finer stratification.

A mother could have several children in our cohort, and we took this into account in the statistical analysis by using robust standard errors. We adjusted for several relevant covariates chosen a priori based on previously published literature. Information about maternal and paternal age (as a continuous variable), parity (one, two, three and four or more children) and birth year originated from the Danish Medical Birth Registry. We adjusted for parental age and parity since these are risk factors for NTDs and birth year due to the change in the ICD system from ICD-8 codes before 1993 and to ICD-10 codes from 1994. Moreover, we adjusted for maternal socioeconomic factors originating from the Integrated Database for Labor Marked Research at the time of birth of the index child, education in three categories (high, secondary, or primary), income in four quartiles, marital status (married/partner or single) and place of residence (Copenhagen, other cities or rest). We included these to take into account any social gradient in the disease. We had limited information on lifestyle factors, but we did some sub-analysis using data from the Danish National Birth Cohort (DNBC), which contains information about lifestyle factors like folic acid intake and alcohol on about 100,000 women and their offspring [39]. Additionally, we did sub-analysis on smoking in around 940,000 women from the Medical Birth Registry covering the years 1996–2008 and on first-born children only. Furthermore, we stratified by birth year (before and after 1994) to examine bias related to selected induced abortion, which is much more common in the later years. The information on the dispensation of prescriptions was not available until 1995, which was recorded in the Danish Prescription Registry. To account for the possible confounding effects of antidepressant use during pregnancy, we conducted a sensitivity analysis and compared the associations before and after further adjustment for antidepressant use during pregnancy among children born during 1996–2008. Finally, we stratified the main analysis on sex to examine potential effect measurement modification. The number of cases did not support further stratified analysis on separate sex. We used STATA 11 (StataCorp, College Station, TX, USA) for the analyses. We present 95% confidence intervals (95% CI) with all prevalence odds ratios (OR).

### Ethics

This study is based on data collected for other purpose, and we had no individual contact at any point in time with the participating mothers and children. All the analyses were performed on Denmark Statistics using encrypted data. We had approval to perform this study from the Danish Data Protection Agency (J NR. 2008-41-2680) and the local ethics committee (VEK, case number M-20100252) in the Central Region of Denmark. According to the Danish law it is not mandatory to obtain informed consent to conduct register-based research.

## Results

Of the 1,734,190 included children, 34,407 had mothers exposed to antenatal bereavement. During the 30-year follow-up, 889 children had spina bifida, 85 had anencephaly, and 164 children had encephalocele. Bereaved mothers tended to be older, smoke more during pregnancy, and have a higher parity, in comparison to non-bereaved children. (Table 1).

**Table 1:** Baseline characteristics of the study population (%).

Variables	Bereaved (n = 34,407)	Non-bereaved (n = 1,699,783)
**Gender**		
Boys	17,542 (51)	871,875 (51)
Girls	16,865 (49)	827,908 (49)
**Maternal age**		
≤26	10,421 (30)	601,986 (35)
27–30	10,214 (30)	535,201 (31)
31–35	8,404 (24)	366,921 (22)
36–39	4,601 (13)	170,320 (10)
≥40	767 (2)	25,355 (2)
**Paternal age**		
≤26	5,817 (17)	336,833 (20)
27–30	8,821 (26)	482,238 (28)
31–35	9,202 (27)	441,145 (26)
36–39	6,685 (20)	283,732 (17)
≥40	3,227 (9)	121,485 (7)
Missing	655 (2)	34,350 (2)
**Parity**		
First	12,418 (36)	796,192 (47)
Second	13,967 (41)	650,585 (38)
Third	6,137 (17)	202,943 (12)
Fourth or above	1,885 (5)	49,884 (3)
Missing	0 (0)	179 (>0)
**Birth year**		
1978–1993	15,998 (47)	852,914 (50)
1994–2008	18,409 (53)	846,869 (50)
**Maternal education**		
High	10,594 (31)	479,224 (28)
Secondary	11,255 (33)	565,412 (33)
Primary	11,986 (35)	612,747 (36)
Missing	572 (2)	42,400 (2)
**Maternal Income**		
No income	550 (2)	34,001 (2)
Low	7,161 (21)	344,972 (20)
Middle	13,394 (39)	650,609 (38)
High	12,967 (37)	639,848 (38)
Missing	335 (1)	30,553 (2)
**Maternal marital status**		
Married/cohabitant	16,929 (49)	815,751 (48)
Single	17,143 (50)	853,684 (50)
Missing	335 (1)	30,348 (2)
**Place of residence**		
Copenhagen	8,030 (23)	407,249 (24)
Other cities	4,055 (12)	204,853 (12)
Other	21,987 (64)	1,057,333 (62)
Missing	335 (1)	30,348 (2)
**Maternal smoking during pregnancy**		
Yes	6,070 (18)	225,553 (13)
No	14,298 (41)	682,039 (40)
Missing	14,039 (41)	792,191 (47)

We compared bereaved mothers to non-bereaved mothers and found a no excess risk of NTDs in the offspring (OR = 0.84; 95% CI: 0.53–1.33). When stratifying on sex, a higher risk for NTDs was seen in girls (OR = 1.42; 95% CI: 0.85–2.38), while a significantly decreased risk in boys (OR = 0.27; 95% CI: 0.09–0.85) (p-value for interaction between bereavement and sex of the child was 0.011). When we stratified the exposure according to loss of different types of relatives, timing and causes of death no elevated risk was found (Table 2). Sub-analysis with more details about several lifestyle factors was based on data from the DNBC and the study population was 81,704 women (n exposed = 1,641). Analysis of supplemental intake of folic acid comparing intake in the bereaved mothers (25.2%) to the intake in non-bereaved mothers (24.2%) showed that there was no difference in intake. The ORs remained similar before and after further adjustment for folic acid intake. Also pre-pregnancy alcohol use and alcohol use during pregnancy showed no significant difference between the two exposure groups. Results from sub-analysis where we adjusted for smoking seemed to attenuate the association, but this was based on very few cases. Sensitivity analysis revealed comparable results when we further adjusted for antidepressant use during pregnancy. Moreover we analyzed overall exposure to antenatal bereavement on first-born children and after divided the study population according to birth year before and after 1994 due to the change in the ICD-codes and the results were not altered.

**Table 2:** Odds ratio for all neural tube defects according to different categories of bereavement.

Bereavement	Cases /N	Crude OR	Adjusted OR (95% CI) ^a^
**Non-bereaved**	1, 096/1,699,783	1 (ref)	1 (ref)
**Any loss, any time, any kind**	19/34,407	0.86 (0.54–1.35)	0.84 (0.53–1.33)
**Type of relative**			
Death of a child	3/5,932	0.78 (0.25–2.44)	0.78 (0.25–2.43)
Death of other relatives	16/28,475	0.87 (0.53–1.43)	0.85 (0.51–1.41)
**Cause of death** ^b^			
Sudden death	3/4,877	0.95 (0.31–2.96)	0.95 (0.31–2.94)
Other death	15/29,213	0.80 (0.48–1.32)	0.77 (0.46–1.31)
**Timing of bereavement**			
12–7 month before conception	6/13,801	0.67 (0.30–1.50)	0.69 (0.31–1.55)
6–0 month before conception	6/15,039	0.62 (0.28–1.38)	0.64 (0.28–1.42)
First trimester	7/5,567	1.95 (0.93–4.10)	1.74 (0.78–3.89)

^a^ Adjusted for maternal age, paternal age, parity, birth year, education, income, cohabitation and place of residence

^b^ 317 bereavement with no information on causes of death not included

## Discussion

In this large population-based follow-up study with prospectively collected exposure data, we found that exposure to maternal bereavement up to one year before pregnancy and during the first trimester was not associated with an increased prevalence of NTDs in the live-born offspring. However, we found the association was modified by the sex of the child. Since we only had access to data on live born, fetuses with a congenital neural malformation of any kind that do not survive until birth are not included. Thus, if exposure to bereavement increases the fetal case fatality, that is if pregnancies result in abortion or miscarriage they are not counted in our study of live-born children, an association may be concealed, and a higher mortality in exposed male fetuses could lead to the apparent “protective” effect in this group.

Li et al. found maternal stressful life events like death or severe disease in first-degree relatives to be associated with an increased risk of NTDs (adjusted OR 4.2; 95% CI 1.4–12.6). [29] Suarez et al. found that women exposed to one or more stressful events of either job changes, residential moves or physical injuries were almost three times more likely to have given birth to a child with some form of NTDs in a population of live born, stillborn and medically or spontaneously aborted. [27] Also Hansen et. al showed in a Danish study an increased risk (OR 1.56; 95% CI 1.05–2.27) of malformations originating of the neural cranial crest cells, among them NTDs, in mothers exposed to emotional stress due to death of a close relative or diagnosis of a severe disease in one of the mother’s close relatives. [30] Carmichael et al investigated spina bifida and anencephaly and their relation to stressful events such as job loss, problems or divorce and found about two and a half times greater risk among women who were exposed to emotional stress. [31, 32] The discrepancy to our study may primarily be because we only studied live born children and only had limited cases of anencephaly (85 cases) since most of these ends in abortion or stillbirth. More recent data may furthermore be subject to selective abortions after ultrasound screenings for malformations although this bias may operate in any direction depending on the women’s use of ultrasound screenings after gestational week 19 where these malformations can be detected.

An intriguing finding of our study is that we found a non-significantly increased risk of NTDs in bereaved girls while a reduced risk among bereaved boys. This may be due to selection bias by conditioning on the live children. The fetal mortality rate for male fetuses is higher than that of female fetuses.[40] Therefore, the boys included in the study may be healthier than the girls. The other alternative explanation is chance finding due to multiple testing.

### Strengths and limitations

Our study has several advantages. Firstly, we based the study on prospectively collected data covering all of the Danish population over 30 years from 1978–2008 in Denmark leaving little possibility for recall bias. Secondly, we believe our measure of exposure, maternal antenatal bereavement is causing an emotional reaction followed by a physiological response. [41, 42] Most women, have a close relationship with their relative and would be genuinely affected by the death of a close relative. Finally, we have information on several confounders from the Danish national registries.

Our study also has some important limitations. Firstly, we do not have any information about women having abortions or stillbirth due to NTDs from 1994–2008. Based on data from EUROCAT from 2006 to 2010, approximately 43% of children with NTDs died before they were born either due to natural death or termination of pregnancy because of a prenatal ultrasound scanning for congenital malformations. [43] This presents a challenge to the current study since our study population only address live-borns. In a sub-analysis, we found the percentage of bereaved mothers to be higher among mothers who had previously had an abortion on medical indication after gestational week 20 compared to the rest of the mothers in the study population. Regrettably, we do not have data on the reason for the medical abortion. Thus we cannot know if the abortion was due to congenital malformation or was done for other reasons. Bereaved mothers may be more likely to choose abortion, which would underestimate the risk of NTDs. However, we also compared bereaved mothers to not bereaved mothers regarding their use of screening for malformation in the child during pregnancy (ICD-10 codes: Z36.3) and found that bereaved mothers were more likely to be screened. This may indicate that mother who lost a relative may be more preconscious or worried about the offspring or in closer contact with the health authorities and therefore more often seek advice on health questions of the offspring. It would only generate a bias to our results if the bereaved mothers were more likely to abort a child due to NTDs, which is plausible given her personal grief. We assessed whether children born in the early half of the study period, where there was limited access to ultrasound screenings for malformations, had a higher risk of NTDs, but dividing the time period did not alter the results significantly. Moreover, mothers having experienced an abortion and then getting pregnant shortly after may also experience a significant amount of emotional stress. The distance between the pregnancies may indicate that some mothers try to cope with losing a child perhaps due to congenital malformations by becoming pregnant shortly after a child loss. This may be seen as a self-treatment by conceiving a replacement child for the child that died and may lead to bias if the already bereaved mother has a greater risk of conceiving a child with a NTDs compared to non-bereaved mothers. To examine this we analyzed if overall bereavement in first-born children was associated with NTDs and this did likewise not alter the results. Secondly, even with a large study population, our study may not have sufficient power to detect a moderate effect if it exists. On the other hand, we performed multiple comparisons and the risk of chance findings increases with numbers of comparisons. Some of our findings may be due to chance. Our stressor of bereavement may act in a context-dependent manner, and may be influenced by lifestyle, cultural, and social factors. Our results may be specific to Denmark or perhaps the Nordic countries.

## Conclusion

We found maternal bereavement in the antenatal period not to be related to the prevalence of NTDs in the live born offspring. New studies could include data from abortions after the ultrasound prenatal screening, and stillborn children.
